# Reviewed article: Carvalho JB, Salviano AF, Lourenço BH, Tomita LY. Temporal trend of food consumption indicators of adolescents monitored by Primary Health Care services of the Brazilian National Health System, 2015-2019. Epidemiol Serv Saude. 2025:34;e20240893

**DOI:** 10.1590/S2237-96222025v34e20240893.b

**Published:** 2025-08-04

**Authors:** Luana da Silva Baptista Arpini

**Affiliations:** Instituto Capixaba de Ensino Pesquisa e inovação (ICEPi)

**Table:** 

Rating	Excellent	Good	Average	Below average	Poor
Interest	✓				
Quality		✓			
Originality	✓				
Overall		✓			

**Table:** 

Questionnaire	Yes	No	Not applicable
Does the manuscript contain new and significant information to justify publication?	✓		
Does the Abstract (Summary) clearly and accurately describe the content of the article?	✓		
Is the problem significant and concisely stated?	✓		
Are the methods described comprehensively?	✓		
Are the interpretations and conclusions justified by the results?	✓		
Is adequate reference made to other work in the field?	✓		

## Manuscript Structure

Length of article is: Adequate

Number of tables is: Adequate

Number of figures is: Adequate

Please state any conflict(s) of interest that you have in relation to the review of this paper (state “none” if this is not applicable): None

## Comments

Prezados autores, parabéns pelo trabalho desenvolvido.

O tema é relevante no meio acadêmico e embora o trabalho esteja bem desenvolvido, tenho alguns comentários que visam aprimorá-lo para a publicação.

Recomendo fortemente que haja revisão da escrita (gramatical e ortográfica) do texto completo. Respeitando a estrutura do texto, especifico as minhas sugestões, conforme documento anexo.

